# Understanding Human–Nature Connections Through Landscape Socialization

**DOI:** 10.3390/ijerph17207593

**Published:** 2020-10-19

**Authors:** Li-Pei Peng

**Affiliations:** Department of Bio-Industry Communication and Development, National Taiwan University, Taipei 10617, Taiwan; lipei@ntu.edu.tw; Tel.: +886-2-3366-2939; Fax: +886-2-2363-5879

**Keywords:** landscape socialization, human–nature relationship, social values, sustainability transformation.

## Abstract

Understanding the landscape socialization underpinning the human–nature relationship is essential because it can contribute to assisting us to reconnect with nature. Reconnecting to nature is increasingly recognized as positively contributing to health and well-being. This study aimed to understand people’s connections with nature through landscape socialization under different land use policies. The study assumed that social values, as perceived by residents, facilitates their landscape socialization. Using a questionnaire measuring sense of community and the Social Values for Ecosystem Services application as analytical tools, the study assessed how residents with varying educational attainment, sense of community, and grounded occupation differ in identifying with conservation- and recreation-oriented policy interventions. The results demonstrated the role of landscape socialization in how people connect with nature, and the landscape socialization as a result of long-term policy interventions may exert substantial effects on residents’ social values across various spatial scales. The results deepen the general understanding of system leverage points for creating inner connections to nature which can aid sustainability transformation.

## 1. Introduction

People are increasingly recognizing that reconnecting to nature positively contributes to individual and social health and well-being [1], particularly with regard to psychological and physiological benefits [2]. For example, being connected with nature helps people exercise better, promote the physical health, increase the local knowledge, have a heightened sense of place and cultivate an intuitive respect for ecological conservation. Contact with nature may also prevent mental illness [3]. In this sense, reconnecting to nature benefits to society and people’s social value. The social value can be realized as the benefits created for society and accruing to society as a whole [4]. It can be described as the perceived, nonmarket values, public ascribes, and assessable for various groups [5]. In general, human–nature connections over the long-term affect social value.

Although socio-ecological systems are complex, reconnecting people with nature can help move society towards sustainability [6]. Moreover, natural settings such as landscapes provide opportunities for interpersonal interaction and strengthening connections within communities. Although structural changes are constantly producing public natural landscapes, residents seldom have opportunity to voice their reflections on connections with nature—even if they are commonly saturated by natural settings in their daily lives.

The concept of human–nature connections stems from multiple viewpoints [7]. The most common theory involves the concept of ecosystem services derived from the Millennium Ecosystem Assessment [8]. It emphasizes the benefits of nature to people and quantifies various values of ecosystem services. However, Ives et al. [6] argued that the notion of ecosystem services is insufficient and urged a broader approach to human–nature connections with a discourse tending to “environmental and sustainability challenges across different socio-cultural contexts”. Ives et al. [9] divided the construct of connections to nature or nature connections into five types: material, experiential, cognitive, emotional, and philosophical. Experiential connection is substantially “direct interaction with nature environment”, cognitive connection is “knowledge or awareness of the environment and values towards nature”, and emotional connection is a “feeling of attachment to nature” [6]. It is, basically, an individual analytical scale. Furthermore, understanding connections with nature across various spatial scales of a landscape has become crucial because such an understanding is useful to land and resource managers [10]. People’s perceptions of both nature and policy interventions must not underestimate connections with nature. Moreover, most landscapes near a metropolis are determined by human activities, which are vital to biodiversity conservation and rational land use planning [11]. However, examining how structural changes produced by education or planning policy may be necessary to reveal how human intervention has affected connections to nature.

United Nations Sustainable Development Goal (SDG) 15 of “Life on Land” [12] addresses the commitment to “Leave no one behind” in a coordinated manner. UN Deputy Secretary-General Amina Mohammed stated that “everyone is a development actor” and that “socializing goals” are crucial [13], echoing arguments that equitable access and equal participation are fundamental principles of the SDG era. Identifying the nature connections of residents is related to landscape socialization. From an archaeological perspective, landscape socialization means the “social relationships [of] people [are] constructed with their landscape indicating the relationship between people and their landscape” [14]. It is the “direct social interaction between people and topography where meaning is imbued into the physical features of the terrain by […] human viewers and inhabitants” [14]. The establishment of landscape socialization originates in primary and secondary socialization [15,16,17]. Primary socialization refers to the acquisition of the basic abilities of landscape cognition and appreciation through family and school education, whereas secondary socialization denotes the ability to evaluate landscapes. Residents with a strong sense of community create strong neighborhood bonds. Thwaites [18] stated that residents with a strong bond to their neighborhood are prone to identify, discuss, or criticize the landscapes they encounter. For example, the close relationship between farmers and the land has a decisive effect on farmers’ grounded perception of landscapes when multifunctional agricultural strategies are implemented. Thus, examining how educational attainment, sense of community, and grounded occupation vary with respect to landscape socialization is worthwhile.

The connection of people with nature underpins landscape socialization and is associated with residents’ perceptions of landscapes which are connected with spatial representations of structural change through; for example, policy interventions in land use planning. No matter whether policy interventions are conservation- or recreation-oriented, their influence is becoming increasingly crucial to providing nature connections in urbanized society. This study aimed mainly to understand people’s connections with nature through landscape socialization under different land use policies. Examining landscape socialization, this study aimed to clarify whether educational attainment, sense of community, and grounded occupation (i.e., farmer or nonfarmer) affect residents’ connection to nature under conservation- and recreation-oriented policy interventions. Taking these variables that possibly influence landscape socialization into account, this study hypothesized that a resident with high educational attainment (i.e., above senior or vocational high school), a strong sense of community, and who is not a farmer can better identify conservation- and recreation-oriented social values within the scope of policy interventions compared with a resident with lower educational attainment (i.e., below junior high school), a weak sense of community, and who is a farmer. To elucidate landscape socialization (i.e., the relationship between people and their landscape) [14], this study explored how policy interventions on a landscape (in the past) influence residents’ social value towards that landscape (in the present).

## 2. Methodology

### 2.1. Conceptual Framework

**Figure 1:**
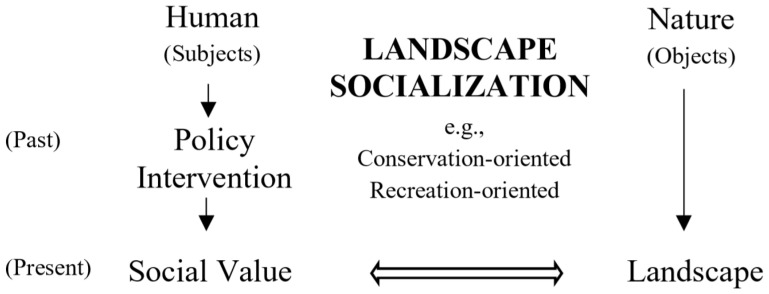
Conceptual framework for understanding human–nature connections.

This study assumes that landscape socialization is measured by a collection of various quantifications of social value that are given by residents (subjects) for specific landscapes (objects). The collection is regarded as representing the comprehensive social value of the landscapes (objects) as a whole. Furthermore, this study analyzed examples involving conservation- and recreation-oriented policy interventions and their corresponding social values. The analytical concept is presented in Figure 1.

### 2.2. Study Area

**Figure 2:**
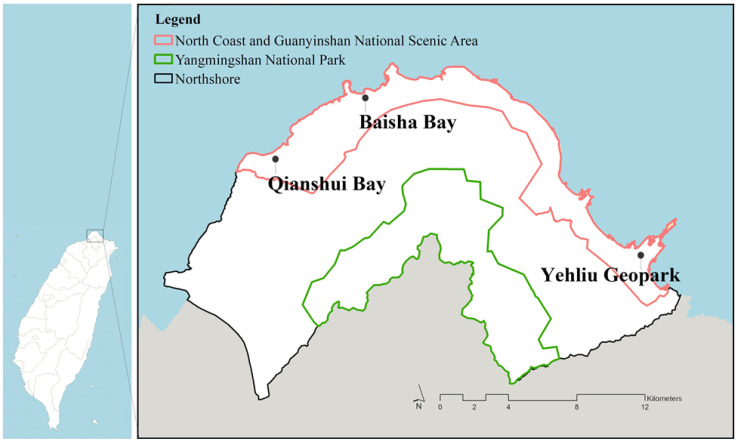
Study location with overlapping scopes of landscape policy.

The north coast of Taiwan, encompassing five administrative districts, was selected as the study site. Landscape policies have been implemented, and these overlap two locations, namely Yangmingshan National Park (YNP, focusing on nature conservation) and the North Coast and Guanyingshan National Scenic Area (NCGNSA, focusing on recreation development). A survey of residents was conducted across the study site, followed by a comparison of differences to assess the stated hypotheses. The north coast has a land area of 300.5 km^2^. The designated area had an overlap of approximately 64.6 km^2^ with YNP and a 61.5 km^2^ overlap with the NCGNSA. The research location and spatial distribution are depicted in Figure 2.

YNP is one of nine national parks in Taiwan. These parks are areas where important natural or historical sites are located. The National Park Law was implemented by the Ministry of the Interior with the objective of achieving sustainable conservation of unique landscapes and ecosystems and the maintenance of biodiversity and cultural diversity. The land planning and operation of parks are organized exclusively by management offices in the Construction and Planning Agency, administered by the Ministry of the Interior. Special attention is given to conservation; no one is permitted to arbitrarily destroy landscapes, damage cultural resources, hunt animals, or pollute the water or air. The National Park Law is strictly enforced by official organizations, with environmental protection as the central tenet to ensure that ecosystems are fully protected. YNP was established in 1985 due to its unique volcanic geology and biological features, and conservation has long been promoted there [19]. After years of conservation advocacy, landscape policies are likely to have intervened in the conservation-oriented landscape socialization of nearby residents.

The NCGNSA is one of 13 national scenic areas in Taiwan. Article 10 of the Act for the Development of Tourism specifies that the objective of these areas is to organize tourism resources within the designated management area to promote them at local and national levels. Governed by the Tourism Bureau of the Ministry of Transportation and Communications, National Scenic Area Administration Offices are responsible for the management of the scenic areas. Land in these areas is not necessarily designated for particular uses, but the objective for the areas is to maintain and encourage local tourism. Accordingly, landscape policies are aimed at promoting the tourism industry on the basis of adequate use of national-level tourism resources within regulatory frameworks. Founded in 2002, the NCGNSA is rich in tourism resources, featuring the world-renowned Baisha Bay, Qianshui Bay, and Yehliu Geopark topographical landscape. Recreational resources are combined with shore and mountain features, diverse natural landscapes, and human-related characteristics [20]. Long-term tourism development has likely enabled surrounding residents to become competent at identifying the recreation-oriented social values of the landscape. Accordingly, this study used YNP for the representation of conservation-oriented social values in landscape socialization, and NCGNSA was used as for the representation of recreation-oriented social values in landscape socialization.

### 2.3. Measures

Using a questionnaire designed to measure sense of community and the Social Values for Ecosystem Services application as analytical tools, this study assessed how residents with varying levels of educational attainment, sense of community, and grounded occupation identify conservation- and recreation-oriented social values of landscape assets.

#### 2.3.1. Background Measurement

To reveal the spatial distribution of the residents’ perceived social values of landscape assets in the north coast area, this study designed a questionnaire to determine residents’ educational attainment, sense of community, grounded occupation, and the social values they attach to the north coast area. Demographic information collected was related to age, occupation, and educational attainment. Age was divided into seven ranges (18–19, 20–29, 30–39, 40–49, 50–59, 60–69, and 70 years or older). Grounded occupation was divided into 10 options (agriculture/forestry/animal husbandry, manufacturing, commerce, service industry, military/public service/teaching, student, homemaker, retired, unemployed, and other). Educational attainment was divided into six options (uneducated, elementary school, junior high school, senior or vocational high school, junior college or university, and graduate school or above).

The design of sense of community indices was based on the Sense of Community Index 2 proposed by Chavis et al. [21], and a 4-point Likert scale (completely disagree = 1, slightly disagree = 2, mostly agree = 3, completely agree = 4) was employed for evaluation. The 24 questionnaire items for sense of community are listed as Table 1. The responses to these items were expected to distinguish residents with a strong sense of community from those with a weak sense of community.

#### 2.3.2. Social Values Measurement

**Figure A1:**
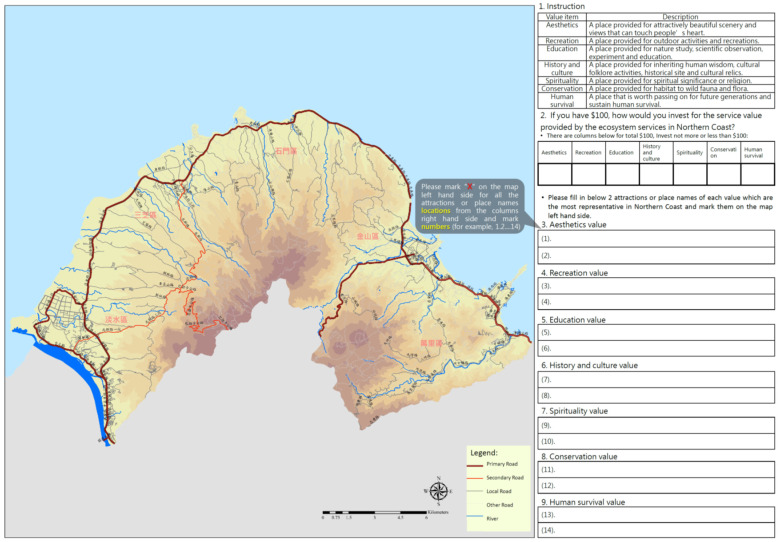
The questionnaire of Social Value.

Residents of local communities can assess the social values that reveal their nature connections through landscape socialization. In addition, because residents understand the social values of landscapes, they should have opportunity to participate in the co-management of landscapes. Consequently, this study presumed that variability in resident characteristics leads to differences in identifying the social values of landscapes—a proxy for landscape socialization in the study area. Spatial ranges designated by the social values of policies as perceived by residents were used to collect and compare residents’ identifications, allowing an understanding of the value, area, and spatial distribution related to the social values of landscapes. For the questionnaire items for social values, the north coast area of Taiwan was selected as the site for identifying positions for spatial analysis. Brown [10] argued that because of the ambiguity of the spatial attributes of landscape value, the social values of landscapes classified in a specific typology comprises constructs that residents can easily associate with landscape attributes when an operational definition is provided. Thus, to help respondents clearly understand the meanings of the elements in the typology, respondents evaluated seven elements of social value—namely aesthetics, recreation, education, history and culture, spirituality, conservation, and human survival—which had operational definitions; this approach is corresponding to scholar’s [22] similar work. A monetary value of NT$100 was allocated across these elements, which reflected how important each element was to the respondent. The questionnaire was written in Mandarin, and the results were subsequently translated into English, as written in Appendix A (Figure A1). This study evaluated the overall weight of the social value elements and subsequently used recreation and conservation as examples to compare the influences of the policy interventions on social values. For each value, the two most representative spatial points were identified, and their names and positions were noted and marked. Basic information such as administrative divisions, road names, and contour lines was integrated into the map so that respondents could locate reference points. In the identification process, only the north coast area was used. Because landscape policy boundaries or areas overlapping with YNP and NCGNSA were neither marked nor mentioned, the identification process was a blind test. 

Although numerous quantitative tools (e.g., InVEST, ARIES, and Envision) have been developed for spatial analysis, quantifying these social values for in-depth analysis required further development [23]. This study utilized Social Values for Ecosystem Services (SolVES 3.0), an open-source toolbar developed by the United States Geological Survey, in combination with ArcGIS 10.4 for quantitative data analysis of landscape social values. Practical cases studies of the social values based on the SolVES model are prevalent [24,25]. In addition, SolVES 3.0 be used to easily obtain and process nonmarket social values. The use of SolVES entailed two basic analytic steps. First, the points identified by the respondents were randomized using an analogous method. The potential point distributions of each social value were then inferred using the quadratic kernel function without considering environmental variables. This provided the intermediate value index, for which a high value indicated a high probability of social value being distributed in a region. 

Second, by applying maximum entropy modelling (MaxEnt), the maximum intermediate value index (Max VI) was used as an input. After the distribution of environmental variables was considered, raster data ranging from 0 to 1 were generated. The closer a value is to 1, the more likely a social value exists under the environmental variable [24]. After these two steps were combined, the SolVES programme outputted a social value index within a value range between 1 and 10 through a standardization process. The obtained value was the maximum score of the social value. For values ranging from 1 to 10, a higher score signifies that a particular social value is more likely to be distributed at a certain location [26].

The study used the geospatial database of the north coast area and ArcGIS for SolVES to analyze the database. After the geospatial data and raster data of various environmental variables were combined, the points identified by the respondents were ordered to create the coordinate data of a digitized space. Based on their questionnaire responses, the characteristics of the residents were analysed. Educational attainment (1-low: below junior high school; 2-high: above senior high or vocational school), sense of community (1-low, 2-high), and grounded occupation (1-farmer, 2-nonfarmer) scores of the respondents were substituted into the human activity subcategory.

#### 2.3.3. Index of Dissimilarity

To analyze respondent variety in terms of two landscape policies, this study overlaid maps of conservation- and recreation-oriented social values onto the spatial ranges of YNP and NCGNSA. The proportion of landscape area and the distribution of two social values in the policy-regulated spatial range of two spaces were presented on maps using ArcGIS. Furthermore, landscape socialization groups were categorized according to demographic variables, namely educational attainment, sense of community, and grounded occupation. Through this grouping method, differences in the spatial distributions of conservation- and recreation-oriented social values were determined. To compare variation among residents with different characteristics, the area of the social value of subcategories (range: 1–10) was calculated using the index of dissimilarity [27]. The formula for the index of dissimilarity is as follows:

(1) $$\Delta =1/2\sum_{i=1}^{n}\left|\frac{ai}{At}-\frac{bi}{Bt}\right|\times 100\%$$

where Δ denotes the index of dissimilarity, *ai* is the area of the *i*-th social value score obtained from the residents of group *A*’s characteristics, *At* is the total area of the social value score of group *A*, *bi* is the area of the *i*-th social value score obtained from the residents of group *B*’s characteristics, and *Bt* is the total area of the social value scores of group *B*.

### 2.4. Survey Implementation

Two areas where natural landscapes have been involved in long-term land use planning were chosen because their landscape boundaries and the scope of policies for the conservation of national parks and the recreation-related development of national scenic areas are clear. A blind test identifying social values was conducted to examine variables (i.e., residents’ educational attainment, sense of community, and grounded occupation) which may indicate the variability of residents’ ability to engage in landscape socialization.

The designated north coast area contains five administrative districts, namely the districts of Tamsui, Sanzhi, Shimen, Jinshan, and Wanli. The area has a total of 89 villages, among which 11 have completed community-based incubation courses in the Rural Regeneration project administered by the Soil and Water Conservation Bureau under the Council of Agriculture, Executive Yuan. After completion of the four-stage (care, advancement, core, and regeneration) incubation courses, these villages could conduct planning and propose projects on their own. These community development associations are located in Jhongshan, Jhongliao, and Shuhsing in Tamsui District; Sanho, Kungjung, Letien, and Ankang in Sanzhi District; Sungshan in Shimen District; Liusan in Jinshan District; and Chungfu and Huangtan in Wanli District. The association members are highly familiar with their residential areas. They participate in bottom-up community affairs, understand the implications of instilling a sense of community, and are familiar with landscape orientation and location, residents’ wording, and relevant contact and policy information [28], all of which enabled them to accurately convey the concept of the survey.

Therefore, members of the 11 community development associations in the aforementioned five districts were recruited as interviewers. Workshops for association members were held at community centers or district offices beginning in July 2017 to give instructions on questionnaire completion and sampling as well as other training. After practice-based skills were developed and hands-on training courses were delivered, 25 people became qualified interviewers who could assist in the investigation of the social values of the policy-regulated landscapes of the north coast area. The formal investigation was conducted in May 2018, when 550 questionnaires were distributed. The interviewers and respondents were given convenience store gift cards as an incentive to increase the response rate. Nonprobability rolling sampling was employed [29] in the hope of deeply engaging with communities and obtaining the opinions of less reachable residents by leveraging local interpersonal relationships between interviewers and residents.

## 3. Results

All 550 questionnaires were returned. Among these 550 questionnaires, 328 were valid and thus retained (valid response rate: 59.6%; remaining landscape socialization points: 1.596).

The data from valid responses were input into Excel. To conveniently calculate the score and rationally present the ranges for one of the variables, namely sense of community, the original range was changed from 1–4 to 0–3, with a maximum total score of 72. The variable sense of community was analyzed using SPSS 18.0 software; descriptive statistics (Table 2) revealed no significant variations between the median (median = 56) and mean scores (mean = 53.89; standard deviation = 12.62; minimum = 9; maximum = 72). This measure was very reliable: the Cronbach’s alpha value was 0.956, which is consistent with the results of Chavis et al. [21]. Thus, the mean value was set as the threshold to differentiate between residents with weak (*n* = 151) versus strong (*n* = 177) senses of community. Residents who selected agriculture or forestry or animal husbandry as their occupation were defined as farmers (*n* = 66), and those who selected another occupation were defined as nonfarmers (*n* = 262). Although the data slightly skewed toward nonfarmers (skewness = 1.497), the variable had a demographically similar sample population in the same study area.

The study revealed that respondents with high or low educational attainment, a strong or weak sense of community, and a grounded or non-grounded occupation achieved different landscape socialization points. The identification of landscape assets with conservation-oriented social values for the entire area was determined using descriptive statistics. Of the respondents, 180 and 148 had high and low educational attainment, respectively, and the respective numbers of identified socialization points were 247 and 203. In all, 151 and 177 had a sense of community above and below the mean value, respectively, and the respective numbers of identified points were 252 and 198. Farmers numbered 66 and nonfarmers 262, and the respective numbers of identified points were 100 and 350. Regarding the perceived recreation-oriented social values of landscapes in the area, the numbers of residents in each category were the same as those for conservation-oriented social values. The numbers of points identified by residents with high and low educational attainment were 280 and 232, respectively. The numbers of points identified by those with a strong and those with a weak sense of community were 269 and 243, respectively. Farmers and nonfarmers identified 112 and 400 points, respectively. Overall, the distribution of respondents and identified points was considered appropriate.

**Figure 3:**
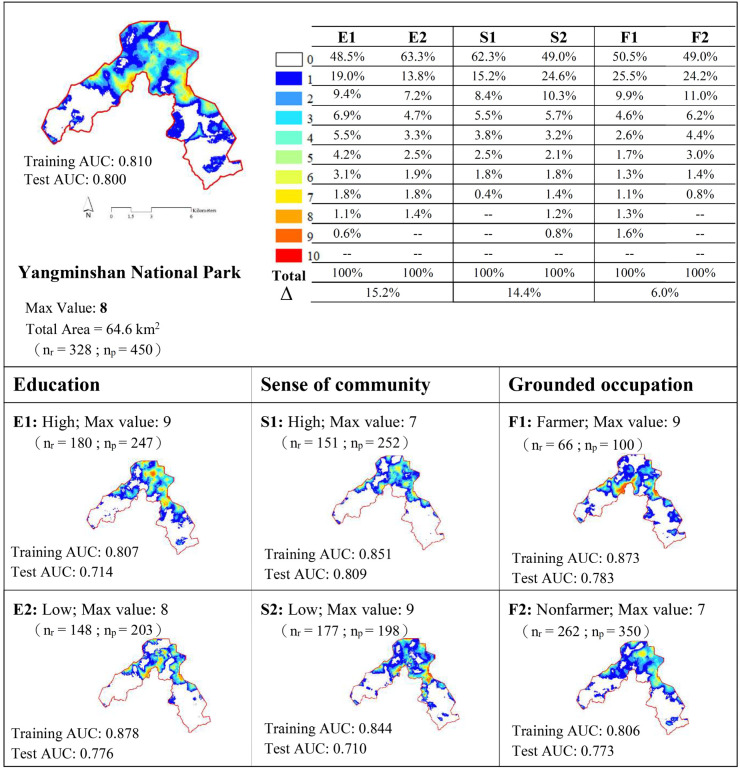
Conservation values between pair assumptions. Note: AUC: area under the curve; %: percentage of total area; Δ: index of dissimilarity; n_r_: number of respondents; n_p_: number of identified points; E1: high educational attainment; E2: low educational attainment; S1: high sense of community; S2: low sense of community; F1: farmer; and F2: nonfarmer.

**Figure 4:**
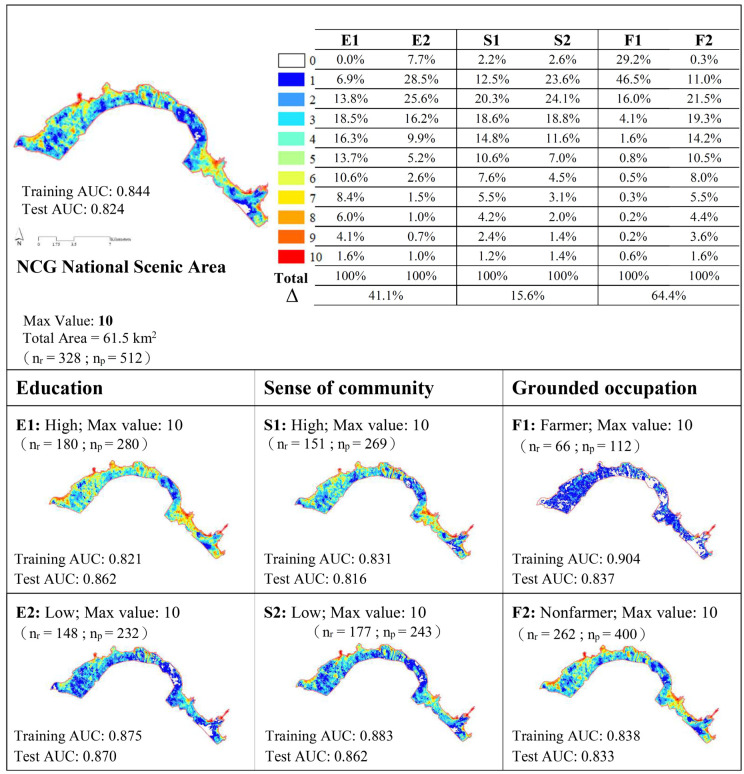
Recreation values between pair assumptions. Note: AUC: area under the curve; %: percentage of total area; Δ: index of dissimilarity; n_r_: number of respondents; n_p_: number of identified points; E1: high educational attainment; E2: low educational attainment; S1: high sense of community; S2: low sense of community; F1: farmer; and F2: nonfarmer.

As Figure 3 and Figure 4 reveal, the values of the area under the curve (AUC) for all samples and for each category of residents were both higher than 0.7 in the training and testing modes of SolVES. The values of the AUC suggested that the models could explain the distribution of identified points and could also be used to predict social values for areas where no primary survey data were available, but which had similar environmental conditions. In terms of conservation-oriented social values, only 47 of the 450 identified points fell within YNP. Regarding recreation-oriented social values, 274 of the 512 identified points fell within the NCGNSA. The maximum score of conservation-oriented social values for all samples was eight. The scores for high and low educational attainment were nine and eight, respectively; the scores for strong and weak sense of community were seven and nine, respectively; and the scores for farmers and nonfarmers were nine and seven, respectively. The maximum score of recreation-oriented social values for all samples and each subcategory was 10. Regarding the identification of conservation-oriented social values, that between high and low educational attainment was 15.2%, that between strong and weak sense of community was 14.4%, and that between farmers and nonfarmers was 6.0%. Regarding the identification of recreation-oriented social values, the index of dissimilarity between high and low levels of educational attainment was 41.1%, that between strong and weak sense of community was 15.6%, and that between farmers and nonfarmers was 64.4%.

Regarding conservation-oriented social values, residents with high educational attainment identified a greater spatial distribution area and had a higher maximum social value score compared with residents with low educational attainment (51.5% vs. 36.7%; 9 vs. 8); the index of dissimilarity between the two groups was 15.2%. In terms of specific spatial distribution, most points identified by residents with high educational attainment were on terrace fields of Sungshan in Shimen District or in the Bayan settlement in Jinshan District, whereas those identified by residents with low educational attainment were on the west side of Datun Mountain and the northeastern side of the Bayan settlement. Residents with a weak sense of community identified a greater spatial distribution area and had a higher maximum social value score compared with residents with a strong sense of community (51% vs. 37.7%; 9 vs. 7); the index of dissimilarity between the two groups was 14.4%. Regarding the specific spatial distribution of the identified points, the groups exhibited a pattern similar to that of educational attainment groups. Farmers and nonfarmers had a similar proportion of landscape socialization distribution, but farmers had a higher social value score than did nonfarmers (9 vs. 7); the index of dissimilarity between the two groups was 6.0%. Regarding the specific spatial distribution of the identified points, most points identified by farmers were on the west side of Datun Mountain and the northeastern side of the Bayan settlement, whereas those identified by nonfarmers were on the terrace fields of the Sungshan community in Shimen District or in the Bayan settlement in Jinshan District.

Regarding recreation-oriented social values, respondents in the high educational attainment group stated that the entire spatial range of the NCGNSA had recreation value (100%). The spatial distribution identified by the low educational attainment group reached 92.3%. The index of dissimilarity between the two groups was 41.1%. Regarding specific spatial distribution, in addition to renowned tourist sites such as Qianshui Bay, Linshanbi, Fugui Cape, the Jinshan seaside area, and Yehliu, residents with high educational attainment identified Jinshan District, Sanzhi District, and other locations in the scenic area and rated them with high scores with respect to proportion of landscape socialization. Residents with low educational attainment gave only 1–3 points to areas other than well-known tourist sites. Groups of residents with strong or weak senses of community identified 97% of the area proportion of spatial distribution, with a 15.6% index of dissimilarity between the two groups. Compared with the group with a weak sense of community, that with a strong sense of community identified more areas of spatial distribution and social value in Jinshan District and Sanzhi District. Regarding grounded occupation, the proportion of areas with spatial distribution identified by nonfarmers was greater than that of farmers (99.7% vs. 70.8%), with a 64.4% index of dissimilarity between the two groups. Compared with farmers, nonfarmers were also more capable of identifying areas with a recreation-oriented social value at renowned north coast tourist attractions, such as Qianshui Bay, Linshanbi, Fugui Cape, the Jinshan seaside area, and Yehliu.

The results revealed that compared with residents with low educational attainment, those with high educational attainment had higher maximum social value scores, and the points identified as having social values were distributed more widely, similar to the current situation. Compared with residents with a weak sense of community, those with a strong sense identified a smaller spatial distribution area and maximum social value score for conservation-oriented policy intervention, but the result for recreation-oriented policy intervention was exactly the opposite. Compared with nonfarmers, farmers had a higher maximum social value score, and the identified points of social values were distributed more widely and precisely in terms of conservation-oriented policy intervention, but the result for recreation-oriented policy intervention was exactly the opposite. Consequently, residents with high educational attainment had a greater understanding of both the national park and the national scenic area, residents’ sense of community did not significantly affect their ability to identify social values, and farmers identified the national park better than nonfarmers did. By contrast, nonfarmers identified the national scenic area more than farmers did.

Notably, regarding the effects of the variables on conservation-oriented social values, the intergroup differences for educational attainment and sense of community exerted greater effects than did grounded occupation. This implies that residents with high educational attainment often absorb, familiarize themselves with, and gain knowledge of landscape policies. Moreover, they may frequently participate in community activities, which increases their ability to absorb information such as that regarding ecological habitats or tourism attractions. Consequently, these people have a higher level of landscape socialization and are familiar with the conservation values advocated by national park landscape policies. Conversely, those with low educational attainment and a weak sense of community rarely participate in community activities. Therefore, they have a vague understanding or lack of understanding of conservation-oriented landscapes, resulting in a discrepancy between the understanding of those with high and low education attainment. Resident education and training and community activities are excellent starting points to increase the perceived value of conservation in landscape policies. Policies and activities related to community construction and rural regeneration, such as policies and experiential activities promoting national park and landscape conservation, can increase residents’ landscape socialization.

Regarding the effects of the variables on recreation-oriented social value, grounded occupation exhibited the highest dissimilarity value, followed by educational attainment and sense of community. This indicates that nonfarmers have a greater understanding of recreation-oriented social values. Compared with residents with low educational attainment, highly educated residents have more experience and knowledge pertaining to tourism and leisure. With a greater understanding of conservation values and actual landscapes, residents with high educational attainment may appropriately comprehend the recreation-oriented landscapes of scenic areas and thus have superior landscape socialization. Residents with low educational attainment might not be able to clearly identify the tourism attractions of scenic areas and rarely—or not at all—believe that their livelihood or daily activities are related to the scenic areas. Therefore, residents with low educational attainment tend to have landscape socialization inferior to those with high educational attainment.

This study has the following limitations. First, because this study adopted only quantitative investigation and spatial analytical methods, specific cases could not be described comprehensively. In addition, this study analyzed only conservation- and recreation-oriented policy interventions and the corresponding social value elements. Therefore, landscape socialization as captured in overall social value or other types of social value could not be analyzed in this study.

## 4. Discussion

A reasonable conclusion from the results is that landscape socialization was indeed an effect of long-term policy intervention, which has accomplished structural changes that have led to perceived social values. Therefore, these effects were reflected in the demographic variables of educational attainment, sense of community, and grounded occupation.

### 4.1. Internalization of Connections to Nature

The results support Kühne‘s [15,16,17] classification concept of primary and secondary socialization. The first uses educational attainment as a factor in analysis. Regardless of the social value identification of conservation or recreation orientation, residents with higher educational attainment are superior at identifying social values; an inference is that the landscape is more socialized. The finding confirms that educational attainment is a crucial factor in landscape socialization. The acknowledgement of landscapes is a subjective process in which opinions vary according to an individual’s life experiences, where they grew up, and their personality traits [30,31,32]. These perspectives highlight the value of the educational attainment of residents, as it may affect landscape socialization and be related to landscape identification. Therefore, this study clarifies that formal education, involving, for example, the natural sciences, experiential activities, and environmental education, is a key variable in landscape socialization.

The study adopted a questionnaire combined with a spatial analysis tool, namely GIS. It assumed that sets of individual cognitions of landscapes can be aggregated to determine the effect of social scale. The results can also be explained as experiential connections which have both spatial and social implications. Although not everyone is equipped with such competencies, social learning through community activities may enhance such capabilities in individuals. Studies [33,34] have revealed that group perspectives such as collective experience, actions, or attitudes towards the environment affect landscape cognition. These perspectives highlight the learning value provided by diverse social groups. A cognitive connection to nature is measured by an individual analytical scale and reflects knowledge of nature [6,35]. In reality, residents strengthen their experiential connections through landscape socialization because they live in local communities and usually have direct experience and interaction with natural landscapes. This also indicates a limitation of this study; it was too brief to include an empirical method for obtaining direct evidence that the results were actually generated from interactions between residents and natural landscapes. Nevertheless, the results are consistent with those of Ives et al. [6], who suggested that “different types of nature connection do not operate in isolation […] (and are) strongly interlinked, especially via emotional and experiential connectedness” [36]. Therefore, landscape socialization may result from the interdependency of experiential and cognitive types of nature connections in the real world.

As for secondary socialization, the results indicate a subtle contrast in the sense of community variable between recreation-oriented and conservation-oriented landscape socialization. Respondents with a strong sense of community have superior landscape socialization in an area covered by a recreation-oriented policy intervention, whereas those with a weak sense of community have superior landscape socialization in a conservation-oriented area. In general, residents who live in a community for a long time have a strong feeling of attachment to or empathy towards nature; sense of community and place attachment are also correlated [37]. Thus, a possible explanation for this phenomenon may be that most respondents lived in communities outside the YNP area. Residents with a strong sense of community have greater group cohesion, spend more time engaged in community affairs, and express concern regarding landscape changes within the community but exhibit less recognition of and concern regarding landscapes outside the communal area.

To restate the concept of nature connection type of Ives et al. [6], emotional connection refers to positive feelings towards nature. Recreation-oriented scenic destinations can bring tourism income, convenient transportation, and possibly economic development. They offer employment and daily life in a tourism atmosphere, and thus, residents here can connect with nature with positive emotions. This study posited that residents, regardless of their sense of community, may regard recreation-oriented landscapes as part of community life because they are accustomed to their areas of residence [38]. Although residents’ sense of community did not significantly affect perceptions of recreation-oriented landscape socialization, a closer inspection revealed that residents with a strong sense of community pay more attention to tourism development opportunities, indicating that they have greater comprehension of recreation-oriented landscape socialization. Residents who scarcely engage in community affairs, namely those with a weak sense of community, are less concerned about opportunities for tourism development. Thus, the landscape-related comprehension of residents with a weak sense of community was slightly inferior to that of residents with a strong sense of community; this result was consistent with the findings of Stewart [39]. This insight illustrates the relationship between sense of community and landscape socialization; residents with a high sense of community seem to be easily affected by regional policy interventions, meaning that they have a more acute emotional affinity with nature [40] and a stronger place attachment to natural areas [41]. Compared with those near the national park, people with a strong sense of community (in a tourism atmosphere) have more opportunities to access sightseeing in the national scenic area, and hence have superior landscape socialization. Therefore, landscape socialization has the effect of inducing an inner trend from having a cognitive to emotional connection with nature.

Conversely, respondents with a strong sense of community exhibit weak landscape-related comprehension of policy intervention vis-à-vis the national park, which is for most outside their community; hence, they have weaker landscape socialization. Presumably, few respondents live in the national park. Residents of outside communities also have fewer place attachments and emotional connections to natural environments in the park. This implies that sense of community seems to be limited to the neighborhood scale. Community-oriented people tend to identify with their own inner, familiar landscapes. When social values are located outside the community at the regional scale, they exhibit less care and inferior landscape socialization. Therefore, sense of community may be a meaningful secondary socialization variable which may be used to identify emotional connections between community and region, or, in alternate phrasing, between insiders and outsiders.

Regarding the concept of human–nature relationships, to strengthen the function of landscape socialization, internalizing connections to nature is necessary. This means focusing on improving awareness and direct interactions from the community to the regional scale. This difference of landscape socialization (between community and regional scales) implies that multilevel governance could potentially be applied to landscapes to appropriately connect communities to regions as well as reorganize the political–spatial dimension of environmental governance, thereby achieving effective landscape governance [42]. However, the concept of landscape socialization does not reflect material and philosophical types. Religious and spiritual values may be necessary to explain types of philosophical connection. Future research may determine whether landscape socialization can scale up inner leverage points.

### 4.2. Landscape Socialization: Incorporating Daily Life in Policy Interventions

The landscape socialization described herein involves social value in the context of desired and actual relationships. This implies relational values between people and nature. An interdisciplinary perspective on the benefits and the perceived, nonmarket quantity of social value [4,5] can yield a variety of value concepts. Chan et al. [43] described relational values as those that refers to diverse roots of relevantly new expression. Relational values also appropriately prioritize existing ways of knowing landscapes. Relational values describing the relationships between people and natural places [43] function at either the individual or the collective level. This study’s results are certain consistent with those of Klain et al. [44], who, when comparing relational values between farmers and tourists, reported that farmers shared higher relational values.

Díaz et al. [45] emphasized that nonmaterial contributions, which are subjective, underpin quality of life at the individual and collective levels. People are increasingly realizing that interactions with nature benefit psychological, social, and physical health and well-being. Shared interactions and experiences with natural environments provide opportunities for social interaction and strengthen networks between social groups and communities. Outdoor recreation is also increasingly essential to enhancing accessibility to or contact with nature while daily natural connection is decreasing in an urbanized society. The functions of natural areas have changed from enabling survival to enriching public recreation experiences where nature is valued differently by residents. Sense of community in this study derives from respondents’ feelings about the community collective, and thus it can illustrate the relational values of landscapes through the human collective. The study determined that sense of community reflects more attention to landscape socialization in small-scale, daily life.

In addition, based on Langley’s [14] anthropological construct, this study addressed whether landscape socialization can be a tool for understanding the human–nature relationship on the basis of the frequency of residents’ interactions with the landscape. Maintaining contact with nature and the behavioral space of daily life in the community is essential, and landscape socialization has potential roles in maintaining harmonious relationships and reconnecting people with nature. Díaz et al. [45] contended that culture plays defining roles in all links between people and nature, and the inclusiveness of local communities is essential. Furthermore, mainstream policies are primarily derived from the natural sciences, with less emphasis on social values than on biophysics, and policy formulation does not benefit from insights and tools developed in the social sciences and humanities [45,46]. Changes to landscapes after policy formulation and residents’ understanding and cognition of landscapes and their social values are of importance because both are directly conducive to promoting resident participation in landscape co-management. Thus, the results indicate that sense of community can affect landscape socialization under various policy interventions, and policy intervention should highlight inclusion and participation and incorporate local knowledge and the cultural context of communities during the policy intervention process.

In addition, the results indicate that farmers exhibit superior landscape socialization under conservation-oriented policy intervention, but under recreation-oriented policy intervention, they exhibit inferior landscape socialization. Farmers pay more attention to the productiveness of land than do nonfarmers. Moreover, they do not readily change agricultural land to other land uses and regard landscape processes as a part of their work processes. Land is the basis of agricultural production; farmers cannot grow crops without land, and their livelihood is based on agricultural landscapes. Because they are in close contact with the land on a daily basis, farmers pay particular attention to conservation-oriented landscapes. Farming is a grounded occupation. With respect to occupational differences between farmers and nonfarmers in Taiwan, the daily lives and production of farmers are embedded in the natural environment, whereas nonfarmers focus on pursuing leisure opportunities outside of work, and thus develop greater concern for recreation-oriented landscapes. This restates Chan et al.’s [43] claim regarding relational values between people and nature, in which people’s personal identities are rooted in the long-term care of nature. Rural farmers take interactions between farming and farmland for granted. Regarding cultural identity, some farmers and communities believe their good and well-being are derived from “Shennong” and the “Earth God” as well as their relationship with nature or others. Farmers’ participation in conservation activities or in planning and implementing national parks should be enabled. This study posits that the characteristics of residents are related to their (comprehension of) landscape socialization.

Regarding recreation-oriented policy intervention, the index of dissimilarity between farmers and nonfarmers was 64.4%, indicating that farmers have inferior landscape socialization. A possible explanation is that farmers regard recreation-oriented landscapes as less crucial because their life, livelihood, and land use are less related to such values. Nonfarmers are generally more sensitive to tourism development opportunities. Although they live within the spatial range of such landscapes, they may commute to work in cities. To release stress from work on weekends, nonfarmers often engage in leisure activities which enable them to gain a recreation-oriented comprehension of landscapes. This result also echoes Stotten’s [47] argument that farmers develop their landscape perceptions from their daily lives. A cultural agricultural activity is an ongoing process through which farmers undergo landscape socialization. Stotten [48] stated that participation and increased awareness among farmers can enhance their analytical and critical abilities. Hence, communities mindful of conservation efforts pay particular attention to the actions of farmers and the preservation of agricultural history in an area, whereas farmers calmly face the use of regional landscapes for tourism and related economic benefits.

## 5. Conclusions

This study mainly sought to determine whether landscape socialization is related to long-term processes of policy intervention functions to connect people with nature. The process of landscape socialization can strengthen human–nature connections, combining experiential, cognitive, and emotional aspects. A long-term structural change such as through a policy intervention affecting residents’ landscape socialization may induce a deeper “reconnect people with nature”. The discussed community–region spectrum may be able to scale up at deep leverage points through landscape socialization.

Landscapes affect perceptions after interaction. Landscapes can also be identified through experiential, cognitive, and emotional types of nature connections which then lead to landscape socialization. Educational attainment, sense of community, and grounded occupation are key variables in landscape socialization, and landscape socialization can help strengthen human–nature connections. In addition, improving education, strengthening sense of community, and encouraging young people to work in agriculture may be effective, but short-term training or fostering work may not be. Considering the human–nature relationship, the concept of landscape socialization helps explain the role from external to internal connections on the system leverage. The spatial identification of social values revealed variation in landscape socialization and demonstrated that sense of community and having a grounded occupation are prominent variables. Landscape socialization is closely related to connections with landscapes and involves experiential, cognitive, and emotional dynamics. This result emphasizes that landscape socialization can help transpose more abstract human–nature relationships; analysis of the three variables suggests that shifting leverage points through the connection of accumulated experiences and then strengthening cognitive and emotional connections to move towards sustainability transformation may be possible.

## Figures and Tables

**Table 1:** Measurement items for sense of community.

No.	Measure Items
1	I am vital to this community because I am a part of it.
2	Community members and I value the same things.
3	This community can truly meet the needs of its members.
4	I am very happy to be a member of this community.
5	When I have a problem, I can talk about it with members of this community.
6	Members of this community have similar needs and goals.
7	I can trust members of this community.
8	I know most of the members of this community.
9	Most members of this community know me.
10	This community has symbols and expressions of membership such as clothes, architecture, landmarks, and flags that people can recognize.
11	I devote a lot of time and effort to being a part of this community.
12	Being a member of this community is part of my identity.
13	Fitting into this community is important to me.
14	This community can influence other communities.
15	I care about what other community members think of me.
16	I have influence over what this community is like.
17	If there is a problem in this community, members can solve it.
18	This community has a good leader.
19	Being a member of this community is very important to me.
20	I engage with other community members a lot and enjoy being with them.
21	I expect to be a part of this community for a long time.
22	Members of this community share important events together, such as holidays, celebrations, and disasters.
23	I feel hopeful about the future of this community.
24	Members of this community care about each other.

Source: Chavis et al. [21].

**Table 2:** Descriptive statistics.

Variables	Items	N	%	Min	Max	Mean (SD)	Skewness	Kurtosis
Age	18–19	10	3.0				−0.388	−0.920
	20–29	41	12.5					
	30–39	44	13.4					
	40–49	38	11.6					
	50–59	76	23.2					
	60–69	78	23.8					
	70 years or older	41	12.5					
Grounded occupation	Nonfarmer	262	79.9				1.497	0.244
	Farmer	66	20.1					
Education	Uneducated	30	9.1				−0.291	−1.192
	Elementary school	71	21.6					
	Junior high school	47	14.3					
	Senior or vocational high school	72	22.0					
	Junior college or university	102	31.1					
	Graduate school or above	6	1.8					
Sense of community		328	100.0	9	72	53.89 (12.62)	−0.678	−0.020

## References

[1] Pritchard A., Richardson M., Sheffield D., McEwan K. (2020). The Relationship between Nature Connectedness and Eudaimonic Well-Being: A Meta-analysis. J. Happiness Stud..

[2] Hurly J., Walker G.J. (2019). Nature in our lives: Examining the human need for nature relatedness as a basic psychological need. J. Leis. Res..

[3] Maller C., Townsend M., Pryor A., Brown P., Leger L.S. (2006). Healthy nature healthy people: ‘contact with nature’ as an upstream health promotion intervention for populations. Health Promot. Int..

[4] Phills J.A., Deiglmeier K., Miller D.T. (2008). Rediscovering social innovation. Stanf. Soc. Innov. Rev..

[5] Sherrouse B.C., Semmens D.J. (2015). Social values for ecosystem services, version 3.0 (SolVES 3.0)—Documentation and user manual. US Geol. Surv. Open-File Rep..

[6] Ives C.D., Abson D.J., von Wehrden H., Dorninger C., Klaniecki K., Fischer J. (2018). Reconnecting with nature for sustainability. Sustain. Sci..

[7] Muhar A., Raymond C.M., Van den Born R.J.G., Bauer N., Böck K., Braito M., Buijs A., Flint C., de Groot W.T., Ives C.D. (2018). A model integrating social-cultural concepts of nature into frameworks of interaction between social and natural systems. J. Environ. Plan. Manag..

[8] Millennium Ecosystem Assessment (2005). Ecosystems and Human Well-Being: Synthesis.

[9] Ives C.D., Giusti M., Fischer J., Abson D.J., Klaniecki K., Dorninger C., Laudan J., Barthel S., Abernethy P., Martín-López B. (2017). Human-nature connection: A multidisciplinary review. Curr. Opin. Environ. Sustain..

[10] Brown G. (2004). Mapping Spatial Attributes in Survey Research for Natural Resource Management: Methods and Applications. Soc. Nat. Resour..

[11] Hobbs R. (1997). Future landscapes and the future of landscape ecology. Landsc. Urban Plan..

[12] Wood S.L.R., Jones S.K., Johnson J.A., Brauman K.A., Chaplin-Kramer R., Fremier A., Girvetz E., Gordon L.J., Kappel C.V., Mandle L. (2018). Distilling the role of ecosystem services in the Sustainable Development Goals. Ecosyst. Serv..

[13] Unite Nations New, More Effective Forms of Collaboration among Diverse Actors Essential to Help Vulnerable Groups, Speakers Tell Economic and Social Council Partnership Forum (Meetings Coverage & Press Releases 11 April 2019). https://www.un.org/press/en/2019/ecosoc6970.doc.htm.

[14] Langley M.C. (2013). Storied landscapes make us (modern) human: Landscape socialisation in the Palaeolithic and consequences for the archaeological record. J. Anthropol. Archaeol..

[15] Kühne O. (2009). Grundzüge einer konstruktivistischen Landschaftstheorie und ihre Konsequenzen für die räumliche Planung. Raumforsch. Raumordn..

[16] Kühne O. (2015). The streets of Los Angeles: Power and the infrastructure landscape. Landsc. Res..

[17] Kühne O. (2019). The differentiated socialization of landscape. Landscape Theories.

[18] Thwaites K. (2001). Experiential Landscape Place: An exploration of space and experience in neighbourhood landscape architecture. Landsc. Res..

[19] Liu X., Wang J.Y., Wang Q.F. (2005). Current status and conservation strategies for Isoetes in China: A case study for the conservation of threatened aquatic plants. Oryx.

[20] Lin J.J., Liao R.Y. (2016). Sustainability SI: Bikeway network design model for recreational bicycling in scenic areas. Netw. Spat. Econ..

[21] Chavis D.M., Lee K.S., Acosta J.D. The Sense of Community (SCI) Revised: The Reliability and Validity of the SCI-2. Proceedings of the Paper Presented at the 2nd International Community Psychology Conference.

[22] Petway J.R., Lin Y.-P., Wunderlich R. (2019). Analyzing opinions on sustainable agriculture: Toward increasing farmer knowledge of organic practices in Taiwan-Yuanli Township. Sustainability.

[23] Riper C.V., Kyle G.T., Sutton S.G., Barnes M., Sherrouse B.C. (2012). Mapping outdoor recreationists’ perceived social values for ecosystem services at Hinchinbrook Island National Park, Australia. Appl. Geogr..

[24] Sherrouse B.C., Semmens D.J., Clement J.M. (2014). An application of Social Values for Ecosystem Services (SolVES) to three national forests in Colorado and Wyoming. Ecol. Indic..

[25] Sherrouse B.C., Semmens D.J., Ancona Z.H., Brunner N.M. (2017). Analyzing land-use change scenarios for trade-offs among cultural ecosystem services in the Southern Rocky Mountains. Ecosyst. Serv..

[26] Sherrouse B.C., Clement J.M., Semmens D.J. (2011). A GIS application for assessing, mapping, and quantifying the social values of ecosystem services. Appl. Geogr..

[27] Duncan O.D., Duncan B. (1955). A methodological analysis of segregation indexes. Am. Sociol. Rev..

[28] Peng L.-P., Wang C.-J., Onitsuka K. (2017). Collaborative Conservation of a Socio-Ecological Production Landscape through ICT Tools. Environments.

[29] Biernacki P., Waldorf D. (1981). Snowball sampling: Problems and techniques of chain referral sampling. Sociol. Methods Res..

[30] Piller I. (2001). Identity constructions in multilingual advertising. Lang. Soc..

[31] Yabiku S.T., Casagrande D.G., Farley-Metzger E. (2008). Preferences for landscape choice in a southwestern desert city. Environ. Behav..

[32] Leibenath M., Otto A. (2013). Local debates about ‘landscape’ as viewed by German regional planners: Results of a representative survey in a discourse-analytical framework. Land Use Policy.

[33] Urry J. (2007). Mobilities.

[34] Peng L.P., Hsieh Y.S. (2015). Settlement Typology and Community Participation in Participatory Landscape Ecology of Residents. Landsc. Res..

[35] Nisbet E.K., Zelenski J.M., Murphy S.A. (2009). The Nature Relatedness Scale: Linking Individuals’ Connection With Nature to Environmental Concern and Behavior. Environ. Behav..

[36] Riechers M., Balázsi Á., Abson D., Fischer J. (2020). The influence of landscape change on multiple dimensions of human–nature connectedness. Ecol. Soc..

[37] Fried M. (2000). Continuities and Discontinuities of Place. J. Environ. Psychol..

[38] Baker D.A., Palmer R.J. (2006). Examining the effects of perceptions of community and recreation participation on quality of life. Soc. Indic. Res..

[39] Stewart W.P., Liebert D., Larkin K.W. (2004). Community identities as visions for landscape change. Landsc. Urban Plan..

[40] Kals E., Schumacher D., Montada L. (1999). Emotional affinity toward nature as a motivational basis to protect nature. Environ. Behav..

[41] Stedman R.C. (2003). Is it really just a social construction? The contribution of the physical environment to sense of place. Soc. Nat. Resour..

[42] Görg C. (2007). Landscape governance: The “politics of scale” and the “natural” conditions of places. Geoforum.

[43] Chan K.M., Balvanera P., Benessaiah K., Chapman M., Díaz S., Gómez-Baggethun E., Gould R., Hannahs N., Jax K., Klain S. (2016). Opinion: Why protect nature? Rethinking values and the environment. Proc. Natl. Acad. Sci. USA.

[44] Klain S.C., Olmsted P., Chan K.M.A., Satterfield T. (2017). Relational values resonate broadly and differently than intrinsic or instrumental values, or the New Ecological Paradigm. PLoS ONE.

[45] Díaz S., Pascual U., Stenseke M., Martín-López B., Watson R.T., Molnár Z., Hill R., Chan K.M.A., Baste I.A., Brauman K.A. (2018). Assessing nature’s contributions to people. Science.

[46] Daniel T.C., Muhar A., Arnberger A., Aznar O., Boyd J.W., Chan K.M., Costanza R., Elmqvist T., Flint C.G., Gobster P.H. (2012). Contributions of cultural services to the ecosystem services agenda. Proc. Natl. Acad. Sci. USA.

[47] Stotten R. (2016). Farmers’ perspectives on cultural landscapes in central Switzerland: How landscape socialization and habitus influence an aesthetic appreciation of landscape. Soc. Nat. Resour..

[48] Stotten R. (2013). Kulturlandschaft gemeinsam verstehen—Praktische Beispiele der Landschaftssozialisation aus dem Schweizer Alpenraum. Geogr. Helv..

