# Erratum: Peng, L.-P. Understanding Human–Nature Connections Through Landscape Socialization. *Int. J. Environ. Res. Public Health* 2020, *17*, 7593

**DOI:** 10.3390/ijerph18115738

**Published:** 2021-05-27

**Authors:** Li-Pei Peng

**Affiliations:** Department of Bio-Industry Communication and Development, National Taiwan University, Taipei 10617, Taiwan; lipei@ntu.edu.tw

## Text Correction

The editor and the author would like to make the following corrections about the published paper [1]. The changes are as follows:

Replacing the sentence in “Section 3. Results” in page of 7:

All 550 questionnaires were returned. Among these 550 questionnaires, 328 were valid and thus retained (valid response rate: 59.6%; remaining landscape socialization points: **1.596**).

With

All 550 questionnaires were returned. Among these 550 questionnaires, 328 were valid and thus retained (valid response rate: 59.6%; remaining landscape socialization points: **1596**).
